# Human Insulin Does Not Increase Bladder Cancer Risk

**DOI:** 10.1371/journal.pone.0086517

**Published:** 2014-01-20

**Authors:** Chin-Hsiao Tseng

**Affiliations:** 1 Department of Internal Medicine, National Taiwan University College of Medicine, Taipei, Taiwan; 2 Division of Endocrinology and Metabolism, Department of Internal Medicine, National Taiwan University Hospital, Taipei, Taiwan; 3 Division of Environmental Health and Occupational Medicine of the National Health Research Institutes, Taipei, Taiwan; Innsbruck Medical University, Austria

## Abstract

**Background:**

Whether human insulin can induce bladder cancer is rarely studied.

**Methods:**

The reimbursement databases of all Taiwanese diabetic patients from 1996 to 2009 were retrieved from the National Health Insurance. An entry date was set at 1 January 2004 and a total of 785,234 patients with type 2 diabetes were followed up for bladder cancer incidence until the end of 2009. Users of pioglitazone were excluded and the period since the initiation of insulin glargine (marketed after the entry date in Taiwan) was not included in the calculation of follow-up. Incidences for ever-users, never-users and subgroups of human insulin exposure (using tertile cutoffs of time since starting insulin, duration of therapy and cumulative dose) were calculated and the hazard ratios were estimated by Cox regression.

**Results:**

There were 87,940 ever-users and 697,294 never-users, with respective numbers of incident bladder cancer of 454 (0.52%) and 3,330 (0.48%), and respective incidence of 120.49 and 94.74 per 100,000 person-years. The overall hazard ratios (95% confidence intervals) indicated a significant association with insulin in the age-sex-adjusted models [1.238 (1.122–1.366)], but not in the model adjusted for all covariates [1.063 (0.951–1.187)]. There was also a significant trend for the hazard ratios for the different categories of the dose-response parameters in the age-sex-adjusted models, which became insignificant when all covariates were adjusted.

**Conclusions:**

This study relieves the concern of a bladder cancer risk associated with human insulin. Appropriate adjustment for confounders is important in the evaluation of cancer risk associated with a medication.

## Introduction

Diabetic patients have a significantly higher risk of bladder cancer in terms of incidence and mortality [1]–[3]. However, whether insulin use can be responsible for the diabetes-related bladder cancer is not known, and this has rarely been studied.

In a recent USA case-control study, diabetic patients treated with insulin had an insignificant 2.2-fold higher risk of bladder cancer while compared to subjects without diabetes [4]. However, this study had limitations inherent to its case-control design, and was underpowered with the very small numbers of diabetic patients both in the case group with bladder cancer (diabetic patients: *n* = 66, those treated with insulin: *n* = 7) and in the control group without bladder cancer (diabetic patients: *n* = 25, those treated with insulin: *n* = 7). Furthermore, the study did not differentiate between the different types of diabetes (type 1 diabetes is dependent on insulin therapy) and the different forms of insulin (human insulin or insulin analogs) used, and did not consider the potential confounding effect of diabetes duration (insulin users may also have a longer diabetes duration).

Some *in vitro* and observational studies provided evidence for a potential risk of bladder cancer associated with insulin use. A cross-talk between insulin and epidermal growth factor has been demonstrated in bladder cancer cell lines [5]. Another recent *in vitro* study suggested that high dose human insulin or insulin glargine may promote bladder cancer cell proliferation via phosphatidylinositol 3-kinases-independent activation of Akt [6]. Bladder cancer cells overexpress insulin-like growth factor 1 (IGF-1) [5] and a case-control study in humans showed that patients with bladder cancer have higher level of IGF-1 and lower levels of IGF binding protein-3 than their controls [7].

Insulin glargine, a long-acting insulin analog, has a 6- to 8-fold higher binding affinity to IGF-1 receptor than human insulin [8]. A recent nested case-control study suggested that insulin glargine may increase the risk of all cancers, while human insulin and other types of insulin analogs do not increase cancer risk [9]. Therefore insulin glargine and human insulin may have different effects on cancer development.

Insulin glargine was the first insulin analog introduced into the market of Taiwan in February 2004, but human insulin remains the most commonly used insulin in clinical practice. Therefore, it is clinically important to clarify whether human insulin is associated with bladder cancer. The purpose of the present study was to evaluate whether human insulin use is associated with increased risk of bladder cancer in patients with type 2 diabetes mellitus, by using the National Health Insurance (NHI) databases of Taiwan.

## Materials and Methods

This is a retrospective cohort analysis using the NHI databases including all patients with a diagnosis of diabetes mellitus during the period from 1996 to 2009 in Taiwan.

Since March 1995 a compulsory and universal system of health insurance (the so-called NHI) was implemented in Taiwan. All contracted medical institutes were requested to submit computerized and standard claim documents for reimbursement. More than 99% of citizens are enrolled in the NHI, and >98% of the hospitals nationwide are under contract with the NHI. The average number of annual physician visits in Taiwan is one of the highest around the world, at approximately 15 visits per year per capita in 2009.

The National Health Research Institute is the only institute approved, as per local regulations, for handling the NHI reimbursement databases for academic research. The databases contain detailed records on every visit for each patient, including outpatient visits, emergency department visits and hospital admission. The databases also include principal and secondary diagnostic codes, prescription orders, and claimed expenses. This study was approved by an ethic review board of the National Health Research Institutes with registered approval number 99274.

The identification information of the individuals was scrambled for the protection of privacy. Diabetes was coded 250.1–250.9 and bladder cancer 188, based on the *International Classification of Diseases, Ninth Revision, Clinical Modification* (ICD-9-CM).

We first retrieved the databases of all patients who had been diagnosed as having diabetes and had been under treatment with either oral anti-diabetic agents or insulin during the period of 1996–2009 from the whole nation (*n* = 1,789,776). The selected entry date was 1 January 2004 (before the marketing of insulin glargine, the first insulin analog marketed in February 2004 in Taiwan, to exclude the possible use of insulin analogs in the study).

After excluding patients who had a diagnosis of diabetes after the year 2004 (*n* = 534,522), patients who held a Severe Morbidity Card as having type 1 diabetes (*n* = 5,894, in Taiwan, patients with type 1 diabetes were issued a so-called “Severe Morbidity Card” after certified diagnosis and they were waived for much of the co-payments), patients having a diagnosis of bladder cancer before 2004 (*n* = 7,264), those who died (*n* = 62,176) or withdrew from the NHI (*n* = 9,512) before entry date, duplicated identification number (*n* = 106), unclear information on date of birth or sex (*n* = 5,122), diabetic patients without any reimbursement record after the entry date (*n* = 232,308), patients who had been treated with pioglitazone (*n* = 235,287, to exclude the possible confounding of this drug because it has recently been challenged with a higher risk of bladder cancer [10]–[12], and patients who had been prescribed with insulin only once (*n* = 70,151), a total of 785,234 patients with a diagnosis of type 2 diabetes mellitus and under therapy with oral anti-diabetic agents or insulin were recruited.

Those who had ever been prescribed with insulin (human insulin) before entry date were defined as ever-users (*n* = 87,940, 11.2%); and never-users (*n* = 697,294, 88.8%) were defined as those who had never been prescribed with any insulin before entry date. To evaluate whether a dose-response relationship could be seen between insulin and bladder cancer, tertile cutoffs for the following three variables were used: time since starting insulin in months, duration of therapy in months and cumulative dose in units, were calculated from the databases and used for analyses.

All comorbidities and covariates were determined as a status/diagnosis before the entry date. The ICD-9-CM codes for the comorbidities were [3], [13], [14]: nephropathy 580–589, urinary tract disease 590–599, hypertension 401–405, chronic obstructive pulmonary disease (a surrogate for smoking) 490–496, stroke 430–438, ischemic heart disease 410–414, peripheral arterial disease 250.7, 785.4, 443.81 and 440–448, eye disease 250.5, 362.0, 369, 366.41 and 365.44, dyslipidemia 272.0–272.4, heart failure 398.91, 402.11, 402.91, 404.11, 404.13, 404.91, 404.93 and 428, and cancer other than bladder cancer 140–208 (excluding 188). Medications included rosiglitazone, sulfonylurea, meglitinide, metformin, acarbose, insulin, statin, fibrate, angiotensin-converting enzyme inhibitor and/or angiotensin receptor blocker, calcium channel blocker and non-steroidal anti-inflammatory drugs. Baseline characteristics between ever-users and never-users were compared by Chi-square test.

The incidence density of bladder cancer was calculated for ever-users and never-users and for different subgroups of exposure. The numerator for the incidence was the number of patients with incident bladder cancer during the 6-year follow-up (from 1 January 2004 to 31 December 2009), and the denominator was the person-years of follow-up. For ever-users, the follow-up duration was either censored at the date of initiation of insulin glargine, or bladder cancer diagnosis or at the date of the last record of the available reimbursement databases in individuals without incident bladder cancer. For never-users, the follow-up was censored at the date of insulin initiation (including human insulin or insulin analogs) or bladder cancer diagnosis or the last reimbursement record, depending on whichever occurring first. This ensured no exposure to insulin of any form throughout the whole follow-up period until censor in the referent group of never-users; and no exposure to insulin glargine in the group of ever-users of human insulin.

Cox proportional hazards regression was performed to estimate the hazard ratios for bladder cancer for ever-users versus never-users, and for the various subgroups of dose-response parameters based on the tertile cutoffs. The following two models were created: 1) adjusted for age and sex (age-sex-adjusted model); and 2) adjusted for all variables compared previously as baseline characteristics between ever-users and never-users (full model). To avoid overfitting of the models, the following sensitivity analyses were also conducted. At first, a backward elimination model was used to identify significant variables from all baseline characteristics. These selected covariates were then entered for estimating adjusted hazard ratios for the overall risk and for the various dose-response parameters (“models with selected covariates”).

Analyses were conducted using SAS statistical software, version 9.1 (SAS Institute, Cary, NC). *P*<0.05 was considered statistically significant.

## Results

Table 1 compares the baseline characteristics between ever-users (*n* = 87,940) and never-users (*n* = 697,294) of human insulin. All of the variables differed significantly between the two groups. Ever-users were characterized by older age, higher proportion of female sex, higher proportion with a diabetes duration ≥5 years, higher proportions of all comorbidities and other cancer, and higher proportions of using other medications.

**Table 1:** Baseline characteristics between ever- and never-users of human insulin.

Variables	Human insulin				*P*
	Never-users		Ever-users		
	*n*	%	*n*	%	
*n* = 785234	697294		87940		
Age (years)					
<40	30162	4.33	5011	5.70	<0.0001
40–49	97739	14.02	9293	10.57	
50–59	172061	24.68	17941	20.40	
60–69	188624	27.05	25347	28.82	
≥70	208708	29.93	30348	34.51	
Sex (men)	351798	50.45	41096	46.73	<0.0001
Diabetes duration (years)					
<1	70081	10.05	1423	1.62	<0.0001
1–3	136586	19.59	4661	5.30	
3–5	136423	19.56	7658	8.71	
≥5	354204	50.80	74198	84.37	
Nephropathy	65304	9.37	30430	34.60	<0.0001
Urinary tract diseases	114768	16.46	38595	43.89	<0.0001
Hypertension	351130	50.36	64713	73.59	<0.0001
Chronic obstructive pulmonary disease	86833	12.45	24989	28.42	<0.0001
Stroke	85934	12.32	27378	31.13	<0.0001
Ischemic heart disease	133250	19.11	35536	40.41	<0.0001
Peripheral arterial disease	63911	9.17	24930	28.35	<0.0001
Eye disease	76211	10.93	38694	44.00	<0.0001
Dyslipidemia	274154	39.32	44724	50.86	<0.0001
Congestive heart failure	37020	5.31	15233	17.32	<0.0001
Rosiglitazone	43505	6.24	18771	21.35	<0.0001
Sulfonylurea	490144	70.29	77795	88.46	<0.0001
Meglitinide	37885	5.43	14827	16.86	<0.0001
Metformin	401991	57.65	75406	85.75	<0.0001
Acarbose	42262	6.06	17895	20.35	<0.0001
Statin	131135	18.81	28869	32.83	<0.0001
Fibrate	117057	16.79	24747	28.14	<0.0001
Angiotensin converting enzyme inhibitor/Angiotensin receptor blocker	258472	37.07	58120	66.09	<0.0001
Calcium channel blocker	195830	28.08	46491	52.87	<0.0001
Non-steroidal anti-inflammatory drugs	436770	62.64	80348	91.37	<0.0001
Other cancer prior to baseline	70358	10.09	12044	13.70	<0.0001

Table 2 shows the incidences of bladder cancer between ever-users and never-users of human insulin, and among the different tertiles of dose-response parameters for human insulin exposure. The overall hazard ratio (95% confidence interval) for ever-users versus never-users of insulin was 1.238 (1.122–1.366) in the model after adjustment for age and sex, and was 1.063 (0.951–1.187) in the model after adjustment for all covariates. In the age-sex-adjusted models, ever-users had a significantly higher risk of bladder cancer than never-users, and the risk significantly increased in correspondence to a higher dose of exposure. However, none of the hazard ratios was significant in the full models in either the overall risk comparing ever-users versus never-users or in the dose-response analyses comparing different tertiles of the three parameters of human insulin exposure to never-users.

**Table 2:** Exposure to human insulin and incidences of bladder cancer and hazard ratios comparing exposed to unexposed.

Exposure to human insulin	Case number	Incident bladder cancer	%	Person-years	Incidence rate (per 100,000 person-years)	Age-sex-adjusted model			Full model*		
						HR	95% CI	*P*	HR	95% CI	*P*
Never-users	697294	3330	0.48	3514835.83	94.74	1.000			1.000		
Ever-users	87940	454	0.52	376802.58	120.49	1.238	(1.122, 1.366)	<0.0001	1.063	(0.951, 1.187)	0.2821
**Tertile cutoffs**											
**Time since starting insulin (months)**											
Never-users						1.000			1.000		
<34	28954	140	0.48	124969.58	112.03	1.188	(1.003, 1.407)	0.0464	1.058	(0.887, 1.262)	0.5322
34–67	28996	145	0.50	124194.42	116.75	1.210	(1.025, 1.429)	0.0247	1.060	(0.890, 1.263)	0.5111
≥67	29990	169	0.56	127638.58	132.41	1.310	(1.122, 1.529)	<0.0001	1.069	(0.906, 1.261)	0.4284
* P*-trend								<0.0001			0.3116
**Cumulative dosage of insulin exposure (units)**											
Never-users						1.000			1.000		
<5,000	28591	146	0.51	123848.83	117.89	1.201	(1.017, 1.418)	0.0305	1.112	(0.936, 1.321)	0.2277
5,000–41,070	29448	132	0.45	123113.42	107.22	1.126	(0.946, 1.341)	0.1802	0.954	(0.795, 1.146)	0.6161
≥41,070	29901	176	0.59	129840.33	135.55	1.375	(1.182, 1.601)	<0.0001	1.114	(0.947, 1.310)	0.1944
* P*-trend								<0.0001			0.3133
**Cumulative duration of insulin exposure (months)**											
Never-users						1.000			1.000		
<3.0	29075	140	0.48	124956.92	112.04	1.142	(0.964, 1.352)	0.1253	1.049	(0.880, 1.250)	0.5928
3.0–23.9	28969	132	0.46	121588.25	108.56	1.164	(0.978, 1.385)	0.0875	1.005	(0.836, 1.207)	0.9608
≥23.9	29896	182	0.61	130257.42	139.72	1.393	(1.200, 1.617)	<0.0001	1.121	(0.955, 1.316)	0.1614
* P*-trend								<0.0001			0.2126

* Adjusted for all variables in Table 1.

HR: hazard ratio; CI: confidence interval.

In the sensitivity analyses, age, sex, nephropathy, urinary tract disease, sulfonylurea, metformin and other cancers were significant in the backward elimination model, and they were selected for adjustment as covariates in the “models with selected covariates”. The overall hazard ratio (1.107, 95% confidence interval: 0.995–1.232) and the hazard ratios for the various tertile cutoffs (data not shown) derived from these models were similar to those derived from the full models and they were all not significant. Therefore, the findings in these sensitivity analyses were consistent with the findings observed in the full models (Table 2).

## Discussion

The findings of this large population-based study suggested that exposure to human insulin might appear to be associated with bladder cancer in a dose-response pattern if the models were only adjusted for age and sex (age-sex-adjusted models in Table 2). However, such an association can probably be explained by other important confounders that may be associated with both insulin use and bladder cancer risk (full models in Table 2).

Theoretically, a confounder should be correlated simultaneously with both the exposure (insulin use) and the outcome (bladder cancer), and it should not be an intermediate between exposure and outcome [15]. Besides age and male sex, we have previously identified several important risk factors associated with bladder cancer. These included nephropathy, urinary tract disease and statin use [3]. All of these could exert confounding effects because they are identified risk factors for the outcome of bladder cancer and are also highly correlated with insulin use (Table 1). Therefore a lack of adjustment for these important risk factors in the age-sex-adjusted models (Table 2) would certainly lead to biased estimates showing a significantly higher risk of bladder cancer associated with insulin use.

An important clinical implication of the present study is that, while evaluating the potential risk of bladder cancer associated with a certain medication, adjustment for only age and sex may not be sufficient to infer a cause-effect relationship. The comorbidities such as nephropathy and urinary tract diseases should always be considered for adjustment, because these are important risk factors for bladder cancer and are commonly seen in the diabetic patients who may have a long duration of diabetes and happen to use insulin. Although an *in vitro* study suggested that high dose human insulin may promote bladder cancer cell proliferation [6], the present study did not support that the use of human insulin in clinical practice in the real world would increase the risk of bladder cancer.

Insulin is always used at a late stage of diabetes when pancreatic β cells are exhausted and most oral anti-diabetic agents fail to adequately control blood glucose. Therefore, indication bias may exist when it was used in patients with more comorbidities that may also be linked to bladder cancer. In the present study, we considered most of the important comorbidities and medications as potential confounders. We excluded users of pioglitazone and insulin glargine because pioglitazone had been challenged with a risk of bladder cancer [10]–[12] and insulin glargine may be associated with a higher cancer risk [9]. Residual confounding might not have been completely excluded even if confounding factors were considered for adjustment in the regression models. However, it should be pointed out that whether insulin glargine would increase the risk of bladder cancer can not be answered here and this has to be investigated in future studies.

Smoking is also an important risk factor for bladder cancer [16], but we did not have information of smoking for adjustment and could only consider surrogates that are highly related to smoking, such as chronic obstructive pulmonary disease, ischemic heart disease, stroke and peripheral arterial disease. It is believed that the findings of the present study would not be distorted without the actual adjustment for smoking, because there is no reason to believe that smoking is a major determinant for insulin use.

Animal [17] and *in vitro* [18] studies support a role of IGF in bladder carcinogenesis. Studies in human beings conducted in the USA [7] and in Ireland [19] also provided evidence for such a link between IGF and bladder cancer. In patients with type 2 diabetes mellitus and insulin resistance, hyperinsulinemia as a result of endogenous hypersecretion may activate the IGF pathway, which stimulates cell proliferation and inhibit apoptosis [20]. However, an association with endogenous insulin hypersecretion should not be readily translated into a scenario of the use of exogenous insulin for the control of blood glucose in the diabetic patients.

Although misclassification of bladder cancer might occur, such a probability was low because labeled diagnoses should be printed out in all prescriptions handed to the patients. Mislabeling of a cancer diagnosis would not be acceptable to the patients when they saw the diagnosis. Because the databases were derived from the whole population, there was no concern of potential selection bias related to sampling error.

This study has several strengths. The databases included all claim records on outpatient visits, emergency department visits and hospital admission, and we caught the diagnoses from all sources. Cancer is considered a severe morbidity by the NHI and most medical co-payments can be waived. Furthermore, there is a low drug cost-sharing required by the NHI and patients with certain conditions such as low-income household, veterans or patients with prescription refills for chronic disease are exempted from the drug cost-sharing [21]. Therefore the detection rate of bladder cancer would not tend to differ among different social classes. The use of medical record also reduced the potential bias related to self-reporting.

The study limitations included a lack of actual measurement data for confounders such as obesity, smoking, alcohol drinking, water intake, family history, lifestyle, diet, hair dye use, and some occupational exposure and genetic parameters. In addition, we did not have biochemical data for evaluating their impact. Another limitation is the lack of information on the grading and staging of bladder cancer.

In summary, the present study relieves the concern of a bladder cancer risk associated with the commonly used human insulin. It also points out the importance of appropriate adjustment for potential confounders that may correlate with both insulin use and bladder cancer. However, whether insulin glargine may increase the risk of bladder cancer is an issue awaiting further investigation.
